# The Effect of the Cheneau Brace on Respiratory Function in Girls with Adolescent Idiopathic Scoliosis Participating in a Schroth Exercise Program

**DOI:** 10.3390/jcm13237143

**Published:** 2024-11-26

**Authors:** Anna Badowska, Paulina Okrzymowska, Elzbieta Piatek-Krzywicka, Bozena Ostrowska, Krystyna Rozek-Piechura

**Affiliations:** 1 Centrum Skolioz, 50-088 Wroclaw, Poland; badowska.am@gmail.com; 2 Department of Physiotherapy in Internal Medicine, University of Health and Sport Sciences, 51-612 Wroclaw, Poland; krystyna.rozek-piechura@awf.wroc.pl; 3 Department of Neurology and Pediatrics, University of Health and Sport Sciences, 51-612 Wroclaw, Poland; elzbieta.piatek-krzywicka@awf.wroc.pl; 4 Department of Occupational Therapy, University of Health and Sport Sciences, 51-612 Wroclaw, Poland; bozena.ostrowska@awf.wroc.pl

**Keywords:** adolescent idiopathic scoliosis, conservative treatment, Schroth method, pulmonary function

## Abstract

**Objectives**: The aim of this study was to evaluate the effect of brace use application and the Schroth intervention on lung ventilation and respiratory muscle strength in patients treated long-term with a Chaneau brace and the Schroth method. **Methods**: A total of 26 post-menarche females aged 15.7 ± 1.5 years, with a Cobb angle of 18–48° and a diagnosis of AIS in inpatient rehabilitation were examined. All participants received brace treatment for a minimum of 3 months with a dosage of 20–22 h/day. This study protocol was performed three times: 1—brace intervention—first day of the present study; 2—without the brace—second day of the present study; and 3—Schroth intervention on the same day. **Results**: During the period of brace use, girls treated with a long-term therapy showed significantly reduced values for VC, FVC, and FEV_1_ and significantly higher values for inspiratory muscle strength PI_max_ compared to values obtained in studies without the brace and after single exercises. Expiratory muscle strength did not differ significantly. **Conclusions**: The majority showed restrictive lung ventilation disorders and decreased respiratory muscle strength in relation to norms. There was a significant correlation of PI_max_ with the duration of wearing the brace and the duration of therapy.

## 1. Introduction

Adolescent Idiopathic Scoliosis (AIS), defined as a 3-dimensional deformity of the spine with lateral curvature, changes in the sagittal profile, and rotation in the transversal plane, is the most common type of scoliosis, affecting up to 3% of children and adolescents worldwide [1,2]. Idiopathic AIS in adolescence is the most common, accounting for 80% of all cases [3,4,5].

According to the Scoliosis Research Society (SRS) and Scientific Society on Scoliosis Orthopaedic and Rehabilitation Treatment (SOSORT) guidelines, observation, brace use, exercise, or surgery is recommended for the treatment of AIS, depending on the degree of curvature [6]. At the same time, it is worth noting that AIS can affect psychosocial aspects and patients’ well-being. Patients increasingly report negative effects of AIS on self-esteem and self-worth [6,7]. The aim of conservative treatment is to prevent curvature progression, minimize respiratory complications, and improve quality of life. Critical to optimal therapeutic outcomes are the individualization of the treatment plan and the education of the patient and parent [6].

The study by Rafferty et al. confirms the positive effect of corrective exercises on respiratory functions in patients with AIS [8]. However, according to the current literature, the influence of the brace on thoracic cage mechanics, thoracic cage mobility, and respiratory function is unavoidable. Interestingly, the study by Yagci et al. showed that patients with AIS may have limited pulmonary function due to the brace (while wearing the brace). Therefore, it is worth deepening the current state of knowledge in this field in order to optimize the treatment process of patients with scoliosis [9].

There is no evidence of an association between the degree of curvature and impaired lung function [10,11].

Weinstein et al. studied 631 patients with AIS and found impaired lung function when the initial curvature exceeded 70°. If, by the end of skeletal maturation, the degree of scoliosis reaches critical values, as defined by most authors as between 30° and 50° [12], there is an increased risk of health problems in adulthood, with reduced quality of life, aesthetic distortion, pain, and progressive functional limitation [13]. The changes that occur in the mechanics and ventilation function in patients with AIS can be attributed to the disruption of the thoracic cage caused by vertebral rotation, with a reduction in its sagittal diameter and compliance, which disrupts the normal development of the lungs during the rapid growth period that occurs during adolescence and causes a restrictive ventilation disorder due to the reduction in the space that allows normal ventilation [14,15,16].

Martínez-Llorens et al. and Redding et al. found that rib cage deformity reduces respiratory muscle strength by affecting the location and force generation of the diaphragm and intercostal muscles [17,18]. Flores et al. and other authors confirmed that inspiratory and expiratory muscle strength in AIS was significantly lower than in age-matched healthy individuals and also reported a small correlation between maximum Cobb angle and respiratory muscle strength [19,20,21].

In addition, the studies by Farrell and Garrido indicate that a reduction in the size of kyphosis is a strong predictor of airway narrowing. The studies have shown that the narrowing of the right lower lobe superior segmental bronchus (RB6) is probably caused by its posterior trajectory, which locates it a short distance from the laterally shifted spine. Studies of patients with right-sided idiopathic scoliosis have shown that a reduction in thoracic kyphosis causes a right-sided narrowing of the airway. The severity of hypokyphosis can lead to proximal narrowing of the bronchus intermedius (BI). As the authors indicate, morphological changes in the airways resulting from a deviation in the sagittal plane play a very important role in the deterioration of lung function. The results of the studies indicate the importance of conducting functional diagnostics of the respiratory system in the aspect of treatment process planning [22].

As indicated in the available literature, scoliosis can cause restrictive ventilation disorders associated with a restrictive deformation of the space inside the chest. These changes result from increased stiffness of the chest wall and general impairment of respiratory muscle function [23,24]. The fact of changed respiratory pattern, which causes a limitation of breathing depth, is also important. Initially, exertional dyspnea appears, which progressively evolves into resting dyspnea. These changes are sensitive predictors of restrictive changes in the chest wall [25,26]. It is also important that the reduced mobility of the chest wall due to scoliosis causes a limitation of the respiratory muscles’ effectiveness. As indicated in the literature, the diaphragm thickness fraction (DTF), the diaphragm thickness at the end of maximal inspiration, and the values of FEV1 and FVC parameters are significantly lower in patients with idiopathic scoliosis. It is also important to note that a smaller diaphragm thickness is associated with an increased Cobb angle [27].

Studies by Cimen et al. indicate that the development of thoracic deformation resulting from the development of scoliosis may cause restrictive disorders of the ventilation pattern. The degree of respiratory dysfunction depends on the degree of scoliosis. The authors emphasize that the assessment of respiratory function is crucial, especially in patients with high apical vertebra rotation [28].

The Society on Scoliosis Orthopaedic and Rehabilitation Treatment (SOSORT) advises physiotherapeutic scoliosis-specific exercises (PSSE) and orthotic braces as two of the most common nonoperative treatments for adolescent idiopathic scoliosis [29,30].

PSSE includes individualized exercises which consist of patient education, auto-correction in three dimensions, training in adjusted activities of daily living, and stabilization of the correct posture [31,32].

The Schroth method is one of the most known PSSE interventions [33]. Schreiber et al. described Schroth method exercises as a ‘method that consists of sensorimotor, postural and breathing exercises aimed at recalibration of normal postural alignment, static/dynamic postural control, and spinal stability’ [34]. The therapy plan includes using proprioceptive and exteroceptive stimulation as well as mirror control to improve the patient’s scoliotic posture as well as breathing pattern [35].

Exercises based on the Schroth method are based on corrective breathing exercises based on targeted adjustment of breathing patterns to specific corrective exercises [36].

There are few studies in the literature evaluating the relationship between the size and type of scoliosis and the angle of trunk rotation (ATR) with respiratory function and respiratory muscle strength in subjects with AIS, and the available results are contradictory. There are studies reporting that braces in conservative treatment of AIS can improve pulmonary function after decreasing scoliosis [37] and moreover have no lasting effect on pulmonary function [38]. Other studies report a negative impact of braces on lung function by increasing chest wall stiffness and limiting growth of the lung tissue [9,39]. Therefore, the aim of this study was to evaluate the impact of brace use and single Schroth intervention on respiratory function and respiratory muscle strength in female patients treated long-term with the Chaneau brace and Schroth method. The relationship between respiratory parameters and Cobb angle, trunk rotation angle, and brace wear time in adolescent idiopathic scoliosis were also evaluated. The use of combined therapy in the form of a brace and Schroth exercises improves the functional condition of patients.

## 2. Materials and Methods

### 2.1. Participants

Twenty-six patients with AIS, aged 15.7 ± 1.5 y, with a Cobb angle of 18–48°, participated in this study. The subjects were 100% post-menarche females with a diagnosis of AIS from a local therapeutic rehabilitation center. The inclusion criteria for the participants were diagnosis of AIS by an independent physician and having received conservative treatment—Schroth therapy and bracing (Cheneau brace). Patients were excluded for a history of spine surgery, musculoskeletal or neurological disease, back pain, or any spinal pathology not comorbid with AIS. Medical examinations of all patients showed no history of pulmonary disease and confirmed their good health condition to participate in this study. All participants were non-smokers (self-reported). The biometric characteristics, including scoliotic curvature details, are presented in Table 1.

The experimental protocol was approved by the University Institutional Ethics Committee, and all participants and their parents provided written informed consent for voluntary participation in this study. Before this study commenced, the participants were informed of the procedures and potential risks of the training.

### 2.2. Experimental Procedure

**Figure 1:**
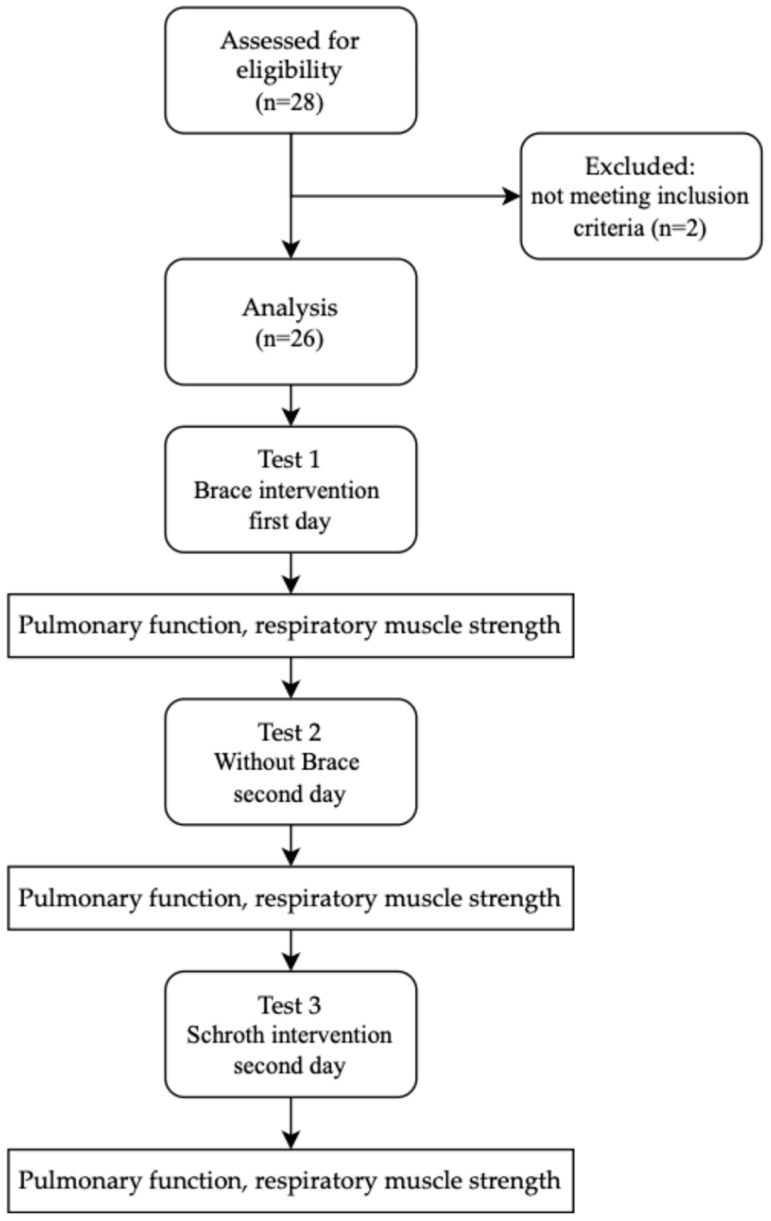
Design and flow of participants through this study.

Twenty-eight patients agreed to participate in this study. The enrollment process is shown in Figure 1.

#### 2.2.1. Test 1—Brace Intervention—First Day of the Present Study

**Figure 2:**
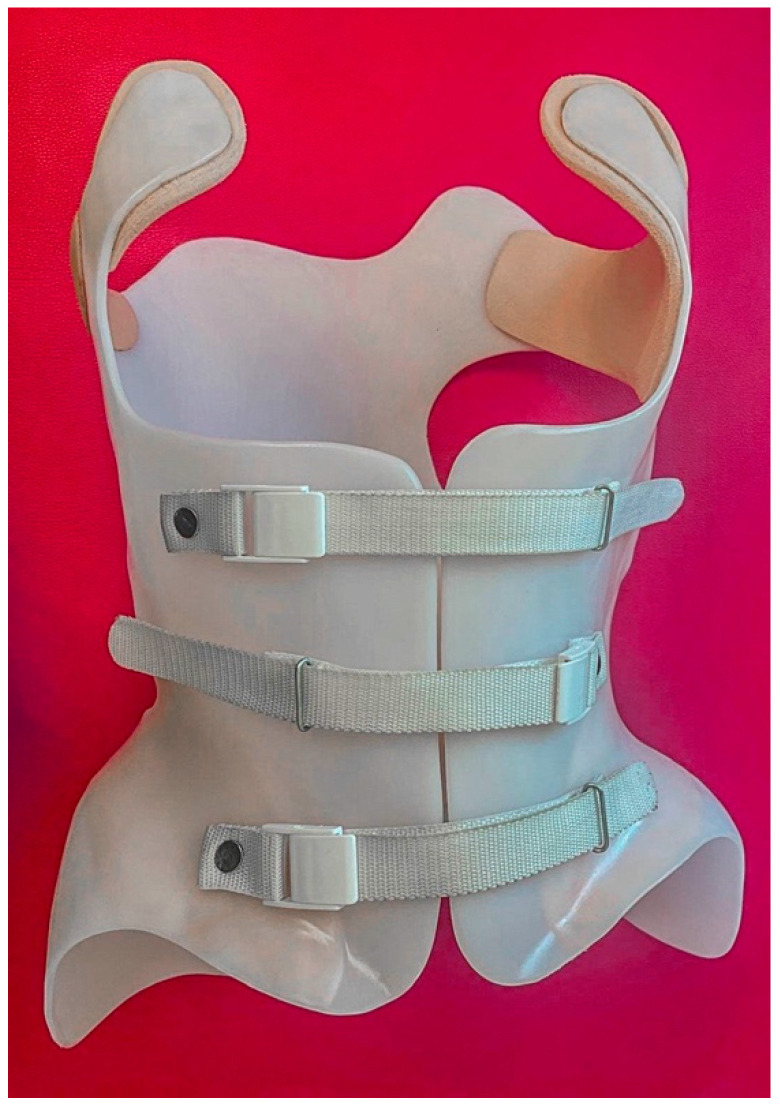
The Chêneau brace used in this study (front).

**Figure 3:**
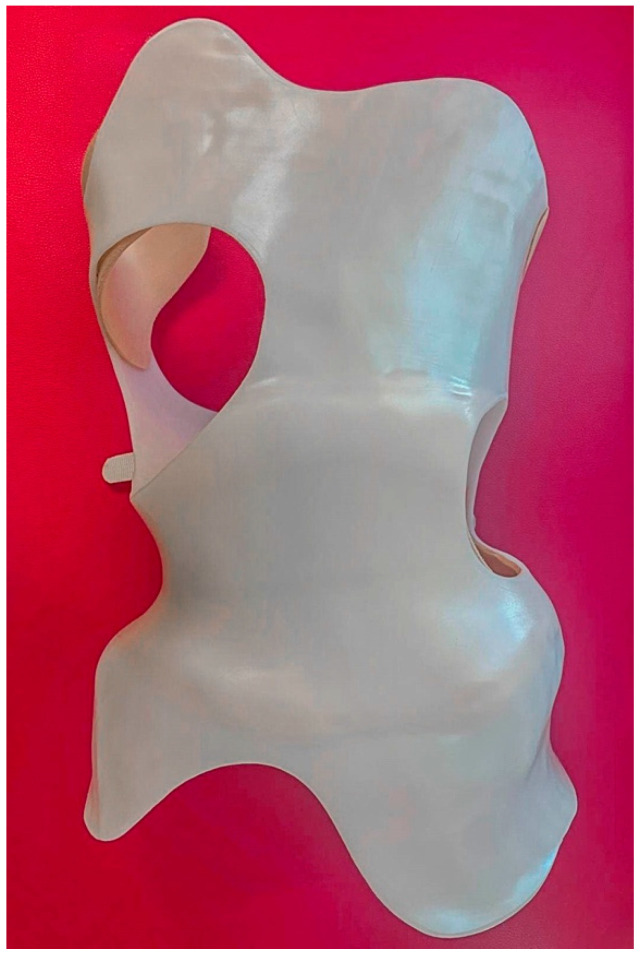
The Chêneau brace used in this study (back).

All participants received brace treatment for a minimum of 3 months with a dosage of 20–22 h/day; the brace (Figure 2 and Figure 3) was removed for personal hygiene, exercise, or delineated rest periods. Each brace was custom designed for three-dimensional curve correction by an experienced orthotics specialist [40]. All participants were informed to not take the brace off 24 h before the experiment.

#### 2.2.2. Test 2—Without the Brace—The Day After the First Test

All participants in this study were informed not to wear the brace for 24 h after the first test.

#### 2.2.3. Test 3—Schroth Intervention—The Day After the First Test

**Figure 4:**
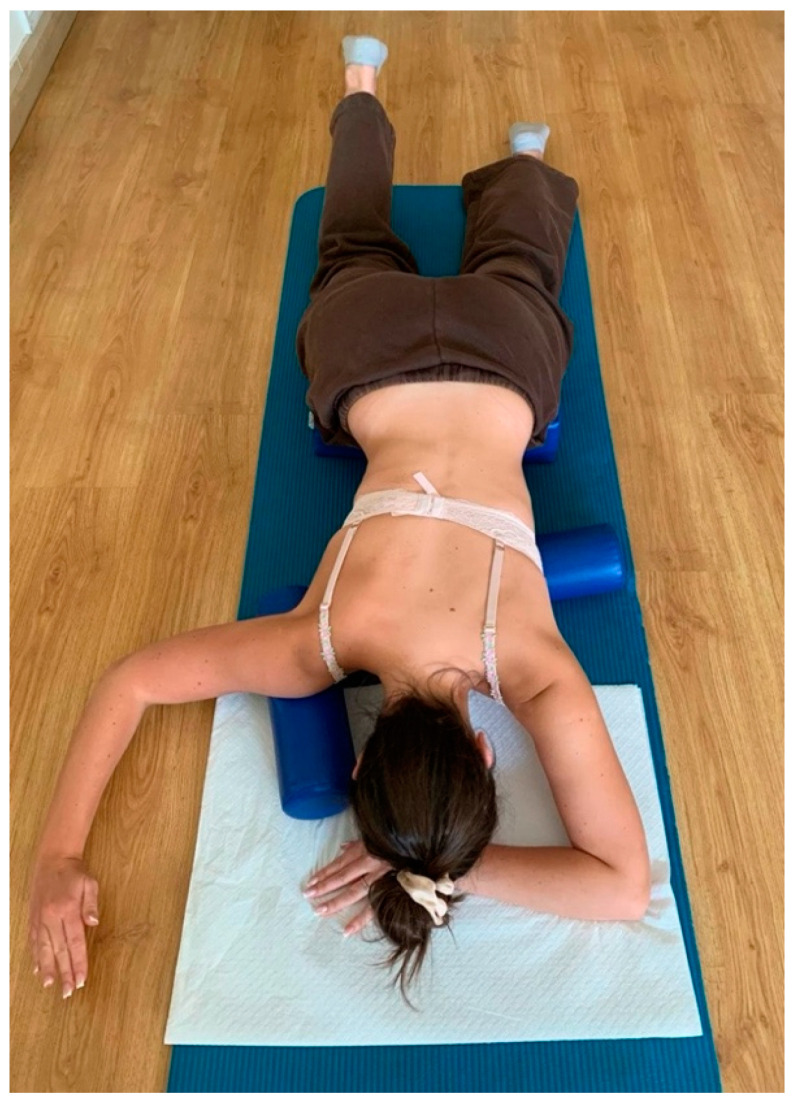
Shoulder counter-traction in prone position.

**Figure 5:**
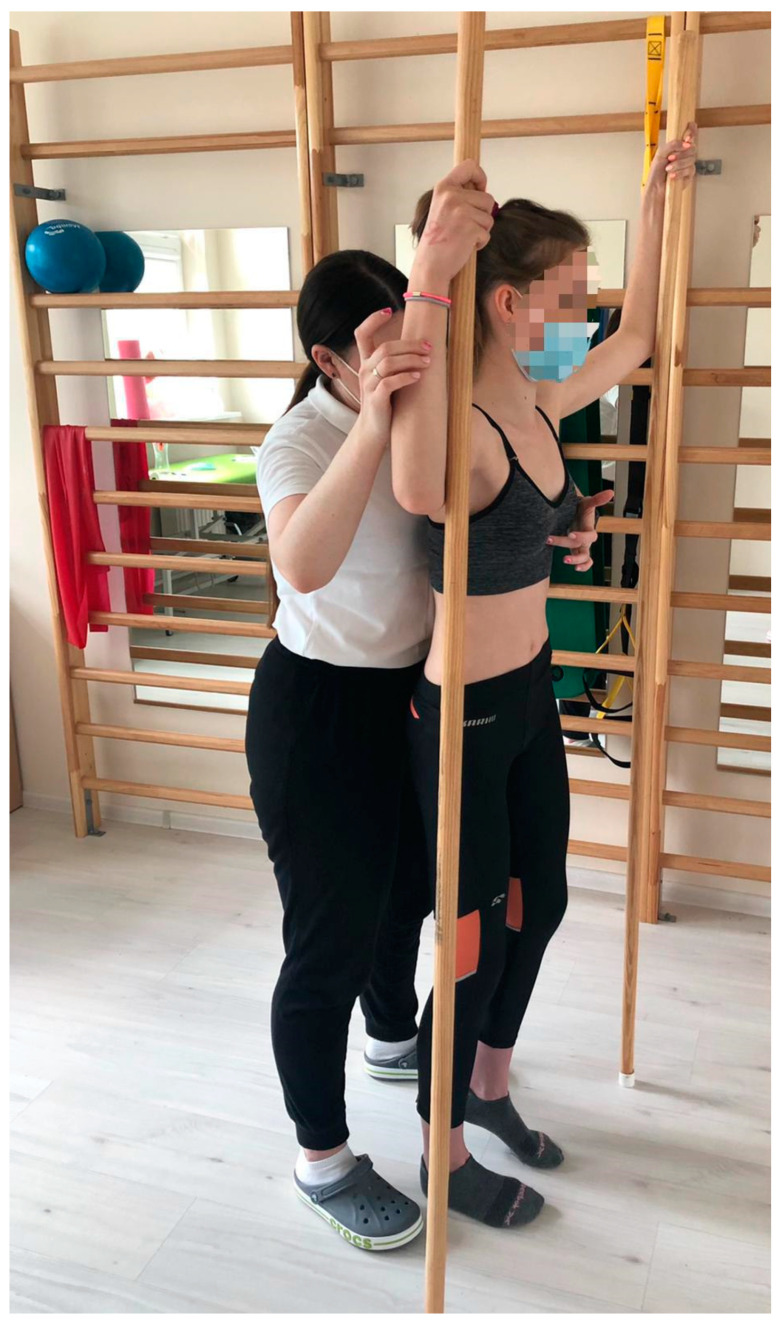
“Between two poles in standing position”.

All participants received supervised Schroth intervention (designed and supervised by a certified Schroth therapist) consisting of a 45-min individual therapy session. The Schroth therapy session consisted of 5 exercises that engaged the muscles of the entire body: basic tension, corrective breathing with resistance, “SCT (shoulder counter traction) in prone position” (Figure 4), sitting “reserved on a chair” with a pole to open the concave part of the curve, and the “between two poles in standing position” exercise (Figure 5). All participants were familiar with the intervention exercises. Participants were executing their exercises in front of the mirror to maintain the quality of the exercises and control the posture. Participants performed 6 reps of each exercise with a 30 s rest between repetitions. A total of 3 sets of each exercise were completed [34].

These types of specific exercises were used to adapt all patients with AIS to the therapeutic program and prevent respiratory dysfunction [31]. Exercises from the Schroth intervention were developed by an experienced international Schroth method instructor. Accordingly, the exercises varied from the elementary exercises in lying positions to exercises in more functional standing positions.

### 2.3. Respiratory Examination

The respiratory function test was performed according to the Standardization of Spirometry—2019 Update [41]—using Flowscreen models 780 and 578, ver. 1.3, Viasys Healthcare, manufacturer Jaeger, Hoechberg, Germany. The spirometer was calibrated according to the manufacturer’s guidelines before the procedure. The respiratory function test procedure was thoroughly explained to the patient. This study was conducted in a quiet and comfortable environment. The room was separate from other patients undergoing this study. The patient wore a nose clip during the test. Additionally, this study uses disposable spirometer filters that are disposed of after the study protocol is completed. During the spirometry test, a flow–volume curve was obtained, and this procedure was repeated three times. The expiratory time during the test had to be at least 6 s, and the measurements of at least two of the three trials had to be repeatable [41].

The following parameters were examined: vital capacity (VC), forced expiratory volume in one second (FEV_1_), forced vital capacity (FVC), the ratio of FEV_1_ to vital capacity expressed as a percentage of VC (FEV_1_%VC), peak expiratory flow (PEF), maximal expiratory flow at 50% of forced vital capacity (MEF_50_), and maximal expiratory flow at 20% of forced vital capacity (MEF20) according to the ATS/ERS guidelines and established reference values [41].

A specialized attachment to the FlowScreen device was used to assess respiratory muscle strength. The maximum inspiratory pressure (PI_max_) and maximum expiratory pressure (PE_max_) exerted by the mouth were assessed [41,42]. During the PI_max_ measurement, patients were instructed to take several breaths through the mouthpiece and then exhale slowly and completely (i.e., to the residual volume). Then, they had to inhale as hard as possible. The patient attempted to maintain the inspiratory effort for at least 1.5 s. The highest negative pressure lasting for at least one second (not a transient spike) was recorded. During the PE_max_ measurement, the subject took several breaths through the mouthpiece. During the actual assessment, the patient, after maximal inhalation (i.e., to the total lung capacity), exhaled as hard as possible. The patient was instructed to maintain the expiratory effort for at least 1.5 s, and the highest positive pressure maintained for at least one second (not a transient spike) was recorded [43].

PI_max_ and PE_max_ for adolescents between 12 and 19 years of age were used to assess the presence of respiratory muscle strength disorders. PI_max_: 71.2–91.6 cm H_2_O and PE_max_: 77.3–102.9 cm H_2_O were considered as normal values for girls. To convert the PI_max_ and PE_max_ values expressed in cm H_2_O into kPa, the formula 1 kPa = 10.2 cmH_2_O was used. The forced vital capacity (FVC) parameter was used to assess the presence of restrictive ventilation disorders. According to the American Thoracic Society (ATS), restrictive disorders are found below 75% of FVC [44]. FVC values up to 60% are defined as mild disorders, values < 60% and >45% as moderate, and values < 45% as severe restrictive disorders [45]. Forced expiratory volume in the first second parameter (FEV_1_) was used to assess the occurrence of obstructive ventilation disorders. According to accepted standards, obstructive disorders are diagnosed below 75% of FEV_1_ [46]. FEV_1_ values up to 60% are defined as mild disorders, <60% and >50% as moderate, and values < 50% as severe obstructive disorders [45].

Respiratory function tests were performed three times for each participant. On the first day, respiratory function tests were performed in a brace (test 1). After the test, the patient was instructed to take off the brace and not wear it until the next day. On the second day, the test was performed twice (without brace). The first test was performed before Schroth intervention (Schroth exercise) (test 2), and the second after the intervention/exercise session (test 3). The interval between the respiratory function test and the therapy was 30 min.

### 2.4. Statistical Analysis

Statistica version 13.3 software was used to analyze the obtained data. The Shapiro–Wilk test was used to examine distributions of variables. ANOVA analysis of variance with the Least Significant Difference (LSD) test was used to compare variables with normal distribution, the significance level was assumed as *p* < 0.05. The results were expressed as X ± SD with 95% confidence intervals. Effect sizes for the magnitude of statistically significant group differences were calculated, and effect sizes were expressed as small at 0.3′, 0.5 is a moderate effect, and 0.8 is a strong effect. Spearman’s rank correlation analysis was used to demonstrate associations.

## 3. Results

**Figure 6:**
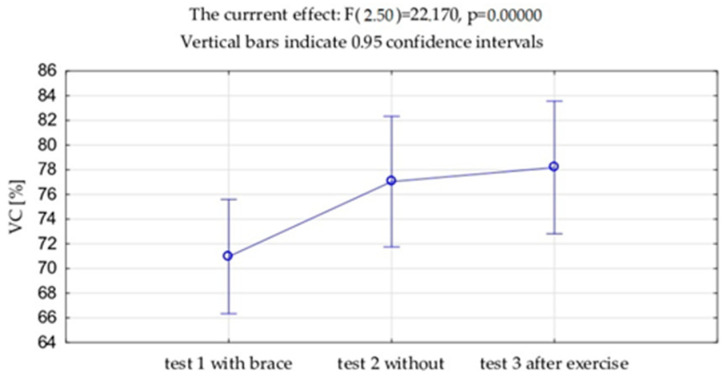
Mean values and standard deviations of the vital capacity (VC) expressed as % of the due value.

**Figure 7:**
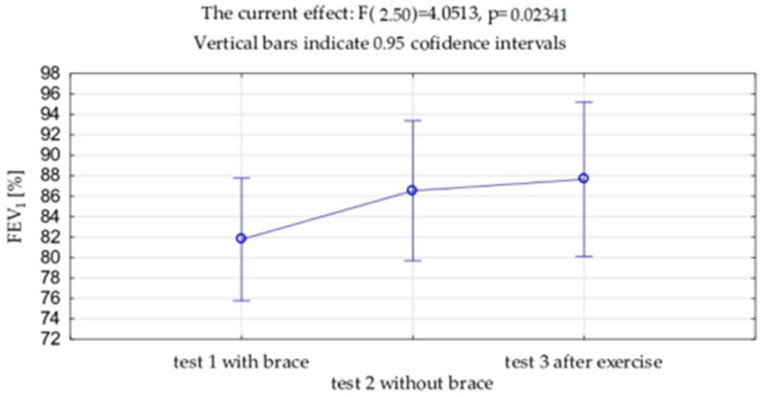
Mean values and standard deviations of the forced expiratory volume in 1 s (FEV_1_) expressed as % of the due value.

VC and FVC expressed in liters and as a percentage of the predicted value showed significantly lower value measured with the brace (test 1) compared to the measurement without the brace (VC *p* = 0.001, FVC *p* = 0.002), both in this study before the exercises (test 2) and after the exercises (test 3) (VC *p* = 0.001, FVC *p* = 0.001). No differences were observed between VC and FVC before and after the exercises. (Table 2, Figure 6 and Figure 7). Exactly the same trends were observed in FEV_1_ expressed in liters and as a percentage of the predicted values.

No significant differences were found between values of FEV_1_%VC, PEF, and MEF_50_ and MEF_25_ indicators.

Interesting results were obtained with respect to the PI_max_ parameter expressing the strength of the inspiratory muscles. A significantly higher value of this parameter was found during the measurement with a brace compared to the measurement without a brace (*p* = 0.028). However, no significant changes in PI_max_ were observed between the measurement with a brace and after the exercises. Equally significant results with respect to the strength of inspiratory muscles were obtained when comparing the measurement before and after the exercises—significantly higher values were measured after the exercises (*p* = 0.009). The same trends were shown with respect to the PI_max_ value expressed as a percentage of the expected values (*p* = 0.002). When analyzing the values of expiratory muscles parameters (PE_max_) measured with and without a brace, no significant changes were observed.

The evaluation of spirometric parameters indicating presence of lung ventilation disorders showed that the VC and FVC parameters expressed as a percentage of predicted values were below 75% and confirmed the presence of restrictive disorders, which is associated with biomechanical changes in subjects with scoliosis. They occurred in 42% of the examined girls, of which 27% had mild disorders and 15% moderate disorders. Measurements with a brace resulted in an increase in the percentage of patients with restrictive disorders to 61.5%, of which 38.5% had mild disorders and 23% had moderate disorders. Measurements carried out immediately after the exercises showed a decrease in the presence of restrictive disorders to only 34.6% of the examined girls, of which 23% had mild disorders and 11.6% moderate disorders.

Moreover, 27% of examined girls with AIS had obstructive disorders, of which 23% had mild disorders and only 4% moderate disorders. The brace and exercises had no impact on the presence of these disorders.

Evaluation of the occurrence of inspiratory muscle strength disorders assessed with PI_max_ [cm H_2_O] showed reduced strength of these muscles measured with a brace in 92.3% of cases. In the other two examinations (without a brace and after exercises), reduced strength was shown in 96.2% of cases (Table 3).

Assessment of inspiratory muscle strength assessed based on PE_max_ value [cm H_2_O] showed reduced values measured with a brace and after exercises in 80.8% of cases. In the examination without a brace, reduced strength was noted in 88.5% of the subjects (Table 3).

Table 4 presents only significant relationships between inspiratory and expiratory muscle strength related to the connection with the duration of Schroth therapy and the time of wearing the brace, which indicates a positive effect of the therapy on respiratory muscle strength. The remaining parameters did not show significant correlations.

## 4. Discussion

In our own studies, significantly lower values of VC and FVC parameters expressed in liters and in percentages of reference values were observed between the examination with a brace and the examination without a brace and before exercises (*p* < 0.001). Similar observations were shown by other authors. Ran et al. observed significant changes in respiratory system function parameters between the group of AIS patients treated with a brace and the control group, in which a brace was not applied. They also observed that the FEV_1_ parameter expressed in liters and the predicted values of FVC (FVC%) and FEV_1_ (FEV_1_%) were significantly lower in the group treated with a brace than in the group without a brace (*p* < 0.05). The observed changes concerned mainly patients with thoracic AIS (*p* < 0.05) [47].

Our own studies also confirm a significant reduction in the FEV_1_ parameter measured with a brace compared to measurement without a brace. A similar relationship was observed in the study by Yurt et al., who showed a reduction in FVC, FEV_1_, PEF, and FEF25-75 values among AIS patients with a brace. The reduction in these parameters may be due to the reduced tension force in the lungs filled with less air. Another explanation for such changes may also be impaired or reduced expiratory muscle strength resulting from the restrictive effect of the brace [38]; our own studies showed a significant reduction in both inspiratory and expiratory muscle strength in most of patients.

In our study, analysis of measurements with a brace showed an increase in the percentage of patients with restrictive ventilation disorders from 42% to 61.5%. The available literature indicates that restrictive changes that increase during the use of a brace result mainly from the limitation of inspiratory movements of the thorax and are a mechanical abnormality of the diaphragm, which is caused by increased pressure in the abdominal cavity [9,38,47].

The decrease in VC and FVC parameters shown in our own studies of girls with AIS without a brace increased the percentage of restrictive ventilation disorders. Other authors also noted a decrease in the values of the same parameters assessed in patients without a brace [9,38,48].

In our study, an important change was observed after the Schroth exercises, which resulted in a reduction in the occurrence of restrictive disorders. Before the exercises, the disorder occurred in 61.5% of patients, while after their performance disorder occurred in 34.6% of the examined patients. This relationship may result from the improvement in the values of VC and FVC parameters after the exercises. Some studies showed a similar observation, which was the result of the improvement in respiratory function under the influence of exercises specific for scoliosis [49,50].

An important change was observed after the Schroth exercises: we observed a reduction in presence of restrictive disorders. Before the exercises, the disorder was observed in 61.5% of patients; while after exercises, it was only observed in 34.6%. This relationship may result from the improvement in the values of VC and FVC parameters after the exercises. Some studies showed a similar observation, which was the result of the improvement in respiratory function under the influence of exercises specific for scoliosis [49,50].

In our own study, a general tendency of restrictive ventilation disorders was observed. Obstructive disorders occurred only in 27% of patients. Assessment of ventilation patterns is extremely important in patients with scoliosis. Interestingly, abnormal breathing patterns and deterioration of respiratory muscle function are reported even in patients with mild scoliosis. Breathing pattern disorders and exercise intolerance are common complications among AIS patients, which cause secondary respiratory muscles weakness and reduced lung capacity [48].

Our own study showed a slight improvement in respiratory function parameters after the exercises, which influenced the presence of restrictive ventilation disorders. The available literature indicates that specific chest stretching exercise programs, including Schroth method techniques, are useful in improving lung function and rib mobility [50]. The improvement in spirometric parameters under the influence of applied exercises may be related to the increased elasticity of the chest walls and the reduction in lung parenchyma compression. Importantly, the Schroth method itself may also support the improvement in the respiratory muscles’ strength [51].

Our own research has shown that wearing a brace reduces the magnitude of the VC parameter and thus increases the incidence of restrictive ventilatory dysfunction. The same observation was seen by another researcher, who showed that brace use adversely affects respiratory function in patients with AIS. Interestingly, the deterioration in respiratory function is independent of the severity or exacerbation of the spinal deformity [52]. The changes occurring in the respiratory system under the influence of the corset may be due to the corrective forces exerted by the brace and its possible restrictive effect on chest expansion during inspiration [47].

Idiopathic scoliosis causes changes in respiratory muscles strength. Our own study showed a tendency towards reduced inspiratory muscle strength among examined patients when tested without a brace. The available literature confirms this trend. The available state of knowledge indicates a general reduction in the strength of inspiratory muscles assessed with the parameter of maximum inspiratory pressure (PI_max_) [14]. Deformations of the thoracic cage cause an increase in its stiffness and a decrease in the strength of inspiratory muscles, which in consequence may cause mechanical failure of the diaphragm. This phenomenon results from an incorrect configuration of the diaphragm. Changes in the position of the diaphragm resulting from deformation of the thoracic cage may cause an increase in its radius of curvature, thus reducing its efficiency and the ability to generate force. At the same time, it is worth emphasizing that a reduction in the PI_max_ value is of great importance in the preoperative diagnostics of patients with scoliosis. A PI_max_ value below 30 cm H_2_O significantly increases the risk of postoperative respiratory failure due to difficult extubation [11]. Our own studies also revealed some girls with a significant decrease in inspiratory muscle strength—below 20 cm H_2_O. This confirms the need for their diagnostics and therapy, especially since a significant correlation was demonstrated between the values of inspiratory and expiratory muscles strength and the duration of therapy.

An interesting observation noted in our own research was the significantly higher strength of the inspiratory muscles generated during the test with a brace compared to the test without a brace. Other authors did not confirm this relationship [9]. In their study, the values of respiratory muscle strength were below the norm, but no significant differences were found in the PI_max_ and PE_max_ values between measurements with and without a brace.

In our study, in relation to the parameter assessing expiratory muscles (PE_max_) measured with and without a brace, no significant difference was observed, and the strength values were low in most cases, confirming their weakness compared to the reference values. Interestingly, other researchers have indicated that the values of maximum expiratory pressure (PE_max_) are usually normal or slightly reduced. Changes in PE_max_ are usually associated with deformation of the chest wall, which does not allow the muscles to work properly and thus generate maximum pressure. According to the literature, changes in lung volume in idiopathic scoliosis are mainly the result of reduced expiratory pressure, which is caused by reduced compliance of the chest wall, impaired lung growth, and impaired overall strength of the respiratory muscles. Due to the ongoing changes, the muscles cannot generate maximum respiratory pressure [14].

Our own research showed a significantly higher value of expiratory muscle strength after the exercises. This may suggest that properly performed Schroth exercises provide good dynamic muscle stabilization. This is confirmed by other authors who pointed out that the Schroth method can support the reconstruction of respiratory muscles by rotating the twisted thoracic cage in the opposite direction through rotational angular breathing (RAB) [51]. Derotation breathing uses the rotation of deformed ribs during exercises [53].

The results of our study showed that patients with mild-to-moderate scoliosis have a predominance of mild pulmonary ventilatory impairment. A similar relationship is also shown in other studies [54]. As the available literature indicates, in mild-to-moderate scoliosis, there may be no clear limitations in respiratory function [54]. As the researchers explain, the mild-to-moderate pulmonary ventilation impairment that occurs may be related to reduced chest wall compliance. At the same time, they emphasize that weakened respiratory muscle strength is also a factor that impairs lung ventilation in patients with mild-to-moderate scoliosis [55]. This trend is also supported by the results of our own study. Interestingly, the results of other studies indicate that limitations in spirometric parameters are not correlated with the degree of spinal curvature impairment in patients with mild and moderate AIS [48], which was also confirmed by the results of our own study.

To sum up, the aim of the present intervention was the assessment of the impact of Schroth exercises on the function of the respiratory system. As a result of these exercises, the increase in ventilation parameter values and the reduction in restriction disorders were measured. Other studies also investigate the impact of this method, not only on posture correction [36], but also on physiopathological changes in the respiratory system caused by scoliosis [56]. According to Liu et al., Schroth exercises provide greater improvements in respiratory function and exercise capacity in AIS patients than aerobic exercises [56]. This study confirms the legitimacy of diagnostic assessment and control of the therapeutic process used in AIS treatment. Further research on larger number of patients, taking into account testing before starting therapy, is an important element of our scientific interests.

The biggest limitation of this study is that it is performed on patients who are already in therapy, and the initial results are not captured prior to the beginning of the therapy. Currently, we have already designed a study that includes initial respiratory diagnostics in AIS patients starting with conservative treatment and periodic assessments of the effects of the therapy. Increasing the size of the group would also be a factor in strengthening the inference.

Clinical implications: restrictive pulmonary impairment is common in patients with AIS and is associated with an increased risk of long-term mortality. Therefore, early detection of pulmonary impairment is very important in reduction severity and associated exercise intolerance through appropriate conservative treatment. Ventilatory dysfunction and skeletal muscle dysfunction are the most important factors limiting the physical capacity of patients with AIS resulting mainly from impaired gas exchange capacity in these individuals.

## 5. Conclusions

During the period of application of the brace, in girls treated long-term with the brace and with the Schroth therapy, significantly decreased values of the VC, FVC, and FEV_1_ parameters were observed, compared to those obtained in the studies conducted without the orthosis and after applying one-time exercises. During the period of application of the brace, in girls treated long-term with the brace and with the Schroth therapy, significantly higher values of muscle strength in the PI_max_ were attested, compared to the studies conducted without the brace and after applying one-time exercises. However, the strength of the expiratory muscles was not substantively different. In the study group, restrictive abnormalities were found in 42% of the patients; measurements in the brace showed an increase in the percentage of patients with restrictive abnormalities to 61.5%, while measurements taken immediately after exercise showed a decrease in the presence of restrictive abnormalities to only 34.6%. The disorders shown were of varying degrees of severity. The majority of the girls studied were characterized by significant weakness of the inspiratory and inspiratory muscles at all three measurement points. There was a moderate correlation of respiratory muscle strength with the time of wearing the brace and the duration of the Schroth therapy in studied girls with adolescent idiopathic scoliosis, indicating the validity of long-term conservative therapy. However, no correlation was observed between respiratory parameters and the Cobb angle and the angle of torso rotation.

Application of adjunctive therapy with use of the brace and the Schroth therapy appears to be a valuable element supporting the physiological functions in girls with adolescent idiopathic scoliosis. The use of conservative treatment using a bracing and Schroth therapy may be an important element in improving the lung ventilation and inspiratory muscle strength of people with AIS and thus their functional fitness. Clinical implications: early detection of pulmonary impairment is very important for the reduction in its severity and associated exercise intolerance through appropriate conservative treatment. Future studies should also include initial respiratory diagnostics in AIS patients starting with conservative treatment and periodic assessment of therapy effects.

## Figures and Tables

**Table 1:** Characteristics of the participants (mean ± SD).

Patients with AIS (*n* = 26)		
**Age [years]**	15.7 ± 1.5	
**Height [cm]**	166.8 ± 6.1	
**Body mass [kg]**	53.6 ± 6.6	
**BMI [kg/m^2^]**	19.3 ± 2	
**AIS curve pattern**	84.6% R thoracic/L lumbar	15.4% L thoracic/R lumbar
**Primary Cobb angle [degrees]**	31.5 ± 7.9	
**Risser sign**	2.7 ± 1.8	
**Schroth therapy [months]**	26.2 ± 19.4	
**Brace treatment [months]**	25.9 ± 19.3	

Abb.: BMI—body mass index.

**Table 2:** Pulmonary functions of the participants with and without the brace and after exercise.

Variable	Test 1with Brace	Test 2Without the Brace	Test 3After Exercise	*p* *	*p* #	*p* ~
	X (SD) (95% CI)	X (SD) (95% CI)	X (SD) (95% CI)			
**VC [l]**	2.6 ± 0.4 (2.4–2.7)	2.8 ± 0.5 (2.6–3.0)	2.8 ± 0.4 (2.6–0.3)	<0.001 * ^a^	0.780	<0.001 * ^b^
**VC [%]**	71.0 ± 11.4 (66.3–75.6)	77.0 ± 13.1 (71.7–82.3)	78.2 ± 13.3 (72.8–83.5)	<0.001 * ^b^	0.269	<0.001 * ^b^
**FVC [l]**	2.7 ±0.5 (2.5–2.9)	2.9 ± 0.5 (2.7–2.1)	3.0 ± 0.5 (2.8–3.2)	0.002 * ^a^	0.414	<0.001 * ^b^
**FVC [%]**	74.4 ± 13.1 (69.1–79.7)	80.9 ± 13.4 (75.4–86.3)	82.3 ± 14.6 (76.4–88.2)	0.002 * ^a^	0.331	<0.001 * ^b^
**FEV_1_ [l]**	2.5 ± 0.5 (2.3–2.7)	2.6 ± 0.5 (2.4–2.8)	2.7 ± 0.5 (2.4–2.9)	0.05 * ^a^	0.492	0.016 * ^a^
**FEV_1_ [%]**	81.8 ± 14.8 (75.7–87.7)	86.5 ± 16.9 (79.6–93.3)	87.6 ± 18.7 (80.1–95.2)	0.05 * ^a^	0.526	0.020 * ^a^
**FEV_1_%VC**	91.3 ± 8.5 (87.8–94.7)	89.2 ± 9.3 (85.4–93.0)	90.4 ± 10.2 (86.3–94.6)	0.203	0.350	0.633
**PEF [L/s]**	4.6 ± 1.1 (4.1–5.0)	4.6 ± 1.1 (4.1–5.0)	4.8 ± 1.4 (4.2–5.3)	0.937	0.283	0.318
**PEF [%]**	67.1 ± 15.6 (60.8–73.4)	67.1 ± 16.0 (60.6–73.6)	69.4 ± 19.4 (61.6–77.3)	0.989	0.351	0.400
**MEF_50_**	3.5 ± 1.0 (3.1–3.9)	3.4 ± 1.2 (2.9–3.9)	3.5 ± 1.2 (3.0–4.0)	0.837	0.439	0.596
**MEF_50_ [%]**	82.9 ± 23.5 (73.4–92.3)	82.2 ± 27.9 (70.9–93.5)	83.0 ± 27.2 (72.0–94.0)	0.838	0.821	0.965
**MEF_25_**	2.1 ± 0.7 (1.8–2.4)	2.1 ± 0.8 (1.7–2.4)	2.1 ± 0.7 (1.8–2.3)	0.725	0.829	0.923
**MEF_25_ [%]**	98.3 ± 35.2 (84.1–112.5)	93.4 ± 40.6 (77.0–109.8)	93.6 ± 31.8 (80.7–106.4)	0.725	0.985	0.288

Abb.: *p* *: comparison between with brace and without the brace; *p* #: comparison between without the brace and after exercise without the brace; *p* ~—comparison between with brace and after exercise; VC: vital capacity; FVC: forced vital capacity; FEV_1_: forced expiratory volume in one second; PEF: peak expiratory flow; MEF_50_: maximum expiratory flow in 50% of forced vital capacity; MEF_25_: maximum expiratory flow in 25% of forced vital capacity. * and bold—significant difference at *p* < 0.05. ^a^ Effect Size > 0.3. ^b^ Effect Size > 0.5.

**Table 3:** Respiratory muscle strength.

	Test 1 with Brace	Test 2 Without the Brace	Test 3 After Exercise	*p* *	*p* #	*p* ~
	X (SD) (95% CI)	X (SD) (95% CI)	X (SD) (95% CI)			
**PI_max_ [kPa]**	4.4 ± 1.7 (3.7–5.1)	4.0 ± 1.6 (3.4–4.7)	4.5 ± 1.7 (3.9–5.2)	0.028 * ^a^	0.009 * ^a^	0.706
**PI_max_ [%]**	73.4 ± 29.4 (61.6–85.3)	67.3 ± 27.2 (56.3–78.2)	73.8 ± 26.9 (62.9–84.2)	0.055 *	0.002 *	0.911
**PE_max_ [kPa]**	6.2 ± 1.8 (5.4–6.9)	5.9 ± 1.7 (5.1–6.6)	6.1 ± 1.7 (5.4–6.8)	0.210	0.368	0.825

Abb.: *p* *: comparison between with brace and without the brace; *p* #: comparison between without the brace and after exercise without the brace; *p* ~—comparison between with brace and after exercise; PI_max_—maximum inspiratory pressure; PE_max_—maximum inspiratory pressure. * and bold—significant difference at *p* < 0.05. ^a^ Effect Size > 0.3.

**Table 4:** Therapy (duration) versus respiratory muscle strength in girls with AIS.

	PI_max_ (Test 1)	PI_max_ (Test 2)	PI_max_ (Test 3)	PE_max_ (Test 1)	PE_max_ (Test 2)	PE_max_ (Test 3)
**Schroth therapy [months]**	0.415	0.370	0.322	0.155	0.453	0.453
**Brace treatment [months]**	0.434	0.381	0.255	0.177	0.406	0.486

## Data Availability

The data presented in this study are available on request from the corresponding author.
